# Participation of the adult population in preventive measures for non-communicable diseases during the COVID-19 pandemic in 2020/2021

**DOI:** 10.25646/10668

**Published:** 2022-12-20

**Authors:** Susanne Jordan, Ronny Kuhnert, Nora Katharina Schmid-Küpke, Anne Starker

**Affiliations:** 1 Robert Koch Institute, Berlin Department of Epidemiology and Health Monitoring; 2 Robert Koch Institute, Berlin Department of Infectious Disease Epidemiology

**Keywords:** PREVENTIVE MEASURES, COVID-19 PANDEMIC, CROSS-SECTIONAL STUDY, POPULATION SURVEY

## Abstract

**Background:**

In 2020/2021, the COVID-19 pandemic and the protective measures associated therewith severely limited the opportunity to participate in prevention and health promotion measures. The article examines the utilisation of the measures and possible factors that are associated with a lower participation during these pandemic years.

**Methods:**

It is based on data acquired between March and August 2021 from the study ‘COVID-19 vaccination rate monitoring in Germany’ (COVIMO), a cross-sectional telephone survey. The data was used to examine the participation in preventive measures in the last 12 months in terms of sociodemographic factors and to analyse a decreased participation with regard to pandemic-related factors. The analysis sample includes individuals aged 18 years and over (n=3,998).

**Results:**

63% of participants generally did not use these programmes, 7% indicated an unchanged participation, 28% reported having participated in fewer measures, and 2% in more measures. Men reported significantly more often than women that they generally do not participate in prevention and health promotion measures. A relevant pandemic-related factor for decreased participation of men was the less clearly perceived comprehensibility of the regulations against the spread of SARS-CoV-2.

**Conclusions:**

Prevention and health promotion should be part of the contingency planning in epidemically significant situations to prevent a decreased participation and to promote health and gender-related equal opportunities even in a crisis.

## 1. Introduction

The prevention and avoidance of non-communicable diseases using structural and individual prevention and health promotion measures is a central task of public health because these diseases represent a high disease burden for the population [1]. The German Prevention Act of 2015 also focuses predominantly on health objectives for the prevention of non-communicable diseases, such as diabetes mellitus type 2 or cancer [2]. The COVID-19 pandemic and the protective measures associated therewith changed the framework conditions for prevention and health promotion of non-communicable diseases. There are still hardly any studies available that use data to show the changes and impacts of the COVID-19 pandemic on prevention and health promotion during the pandemic.

In 2020 and 2021, Germany had partly wide-scale public restrictions during the COVID-19 pandemic with restrictions on movement and contact (so-called lockdown) [3, 4]. This also limited the opportunities for offering or for participating in prevention and health promotion measures for prevention of non-communicable diseases. Group programmes for promoting physical activity, nutritional counselling, or classes for managing stress, did not take place at all at times because institutions, such as adult education centres, fitness studios, sports fields, or gyms were closed. As a result, sports clubs, for example, lost 792,119 members in 2020, a reduction of almost 3% compared to 2019 [5]. Due to enclosed spaces and the temporary obligation to work from home, companies had to limit their health promotion programmes or switch to digital programmes, respectively. Various other providers, such as the statutory health insurance funds, also offered some prevention measures digitally during the COVID-19 pandemic [6]. Nevertheless, the statutory health insurance funds, as most important provider of prevention and health promotion measures, were nonetheless not able to offer approximately one third of their prevention and health promotion measures in 2020 [6]. Compared to the previous year, course participation declined by 36%, and 31% of the health promotion measures could not be carried out in day-care centres, schools, and local communities; in companies it was 36% [6, p. 15, 98]. Due to the reduced number of programmes, an overall decline of the utilisation of (primary) prevention and health promotion measures can be assumed. A pandemic-related decreased utilisation of secondary preventive measures, such as early detection examinations [7] or medical care services has already been shown [8, 9].

Health promotion measures and measures for the prevention of non-communicable diseases are important in times of social crisis, such as the COVID-19 pandemic. Studies were able to show, for example, that for many people, the containment measures for the spread of the pathogen SARS-CoV-2 had a negative impact on the health behaviour, such as physical activity and, associated therewith, also on body weight [10–12]. Negative effects on mental health are meanwhile likewise known in individual population groups [13]. During the pandemic, the health-related and psychosocial effects could be observed more frequently in socially disadvantaged population groups [14], have intensified existing socially-induced health inequality, and suggest pandemic-specific need for support [15, 16].

Information at population level on how widespread the participation in health promotion and prevention measures of non-communicable diseases was in Germany during the pandemic years 2020 and 2021, was not available yet. This article is to close this research gap, and, when answering this first research questions, also takes into consideration whether there were differences within the population with regard to sex, age, and education because these factors were significant for the utilisation of preventive measures even before the pandemic [17, 18]. This refers to programmes directed towards primary prevention, such as courses, exercises, counselling on the topics of diet, physical activity, relaxation, and sport or fitness, which were partly financed by health insurance funds, and which could be hosted by various providers. Secondary preventive measures, such as early detection examinations, are not included.

The article examines a second research question, whether, in addition to the above-described restrictions, there were pandemic-related factors, which led to a decreased participation in certain population groups. In 2020 and 2021, communication about the pandemic was shaped by a variety of sources of information and contents of different quality. In part, contradictory information existed about infection and its containment measures [19]. The resulting uncertainty and difficulties in comprehending information within the population has already been reported in other studies [19, 20] and will be examined here as possible factors on utilisation. This includes (1) the participants’ assessment with regard to uncertainty due to the large amount of information about the COVID-19 pandemic, and (2) the comprehensibility of the rules for the containment of SARS-CoV-2. Additional pandemic-related factors that could be relevant to the decision to participate in a prevention and health promotion measure, are the vaccination status and belonging to a risk group for a SARS-CoV-2 infection or a severe course of COVID-19.

**COVIMO – COVID-19 vaccination rate monitoring in Germany**

**Data owner:** Robert Koch Institute

**Objectives:** Monitoring the willingness and acceptance of different population groups in Germany to get vaccinated against COVID-19.

**Survey methodology:** Interview by telephone at different survey periods (waves), each time with a new sampling (repetitive cross-sectional study)

**Population:** German-speaking population aged 18 and over (exception wave 9, in which 6 languages are recognised)

**Sampling:** Random sample from the sampling system of the ADM (Registered Association of German Market and Social Research Institutes). The sample includes randomly generated mobile and landline numbers (dual frame approach).

**Participants:** Mostly approximately 1,000 individuals for each survey point (wave)

**Response rate:** Depending on the collection period, the response rate is between 24.0% and 27.3%

**Examination period:** January 2021 – December 2022

Further information at www.rki.de/covimo

## 2. Methods

### 2.1 Sample design and study conduct

The data from the study COVID-19 vaccination rate monitoring in Germany (COVIMO) by the Robert Koch Institute was used for the analyses. The primary objective of the COVIMO study is to collect and to analyse the willingness and acceptance of different population groups in Germany to get vaccinated against COVID-19. COVIMO is a repeated cross-sectional study, for which a new random sample is drawn approximately every four weeks from the sampling system of the ADM (Registered Association of German Market and Social Research Institutes) since January 2021 [21]. The sample includes randomly generated mobile and landline numbers (dual frame approach). The data collection takes place using a standardised telephone interview of mostly approximately n=1,000 people of the German-speaking population aged 18 and over. Depending on different survey periods (waves), the response rate for the COVIMO survey is between 24.0% and 27.3% [21]. Additional information about how the study is conducted can be found in the detailed methodology report relating to the study [21].

At four periods during the COVIMO survey (waves), the participation in health promotion measures during the COVID-19 pandemic was additionally collected in the interview. These four COVIMO waves used for the analysis are wave 3 (17.03.2021–10.04.2021), wave 4 (21.04.2021–07.05.2021), wave 6 (28.06.2021–13.07.2021), and wave 7 (26.07.2021–18.08.2021), whereby the total data collection period for the data available here extends from 17.3.2021 to 18.8.2021.

### 2.2 Indicators

#### Participation in preventive measures during the pandemic 2020/2021

The participation in preventive measures during the pandemic was captured using the question: ‘There are a number of health promotion measures that are offered by various providers and which focus, for example, on diet, physical activity, relaxation, and sport or fitness. Such measures are partly financed by health insurance funds. Did you change your participation in such measures (courses, exercises, counselling) in the last 12 months due to the limitations caused by CORONA? Available response options were: (1) ‘No, I do not use such programmes.‘, (2) ‘No, I used the same amount of programmes overall.‘, (3) ‘Yes, I used fewer programmes overall.‘ and (4) ‘Yes, I used more programmes overall ‘. Below, (1) will be referred to as ‘generally no participation’ (or ‘those who generally did not use the measures’), (2) as ‘unchanged participation’, (3) as ‘lower participation’, and (4) as ‘higher participation’.

#### Sociodemographic factors

The evaluations considered the impact of gender. To describe gender differences, the information about gender identity was used in COVIMO: Participants were able to specify, to which gender they felt they belonged (‘male’, ‘female’, ‘diverse’).

Participants’ responses about their age were included in the analyses with four age groups. The four age categories included the following age ranges: 18 to 29 years, 30 to 49 years, 50 to 64 years, and 65 years and over.

The education status was surveyed using the highest level of education and was classified in three education groups: ‘Low education group’: No school-leaving qualification, left school without qualifications, still in school, lower secondary/elementary school graduate, year 9/10 of polytechnic secondary school, school-leaving qualification after attending maximally seven years of school; ‘medium education group’: secondary school level I certificate, general certificate of secondary education, 10th grade of polytechnic secondary school or equal school-leaving qualification; and ‘high education group’: A levels, subject-specific higher education entrance qualification or subject-specific advanced technical college qualification.

#### Pandemic-related factors

To collect the self-assessed uncertainty caused by the large amount and variety of information about the COVID-19 pandemic, the following question was asked: ‘Some people feel uncertain because of the large amount of information about the coronavirus and no longer have any idea what information they can trust. How do you feel: Do you feel uncertain because of the large amount of information?’ This wording, minimally abbreviated, originates from a study by Okan et al. from 2020 und 2021 [19] relating to the health literacy and to the information behaviour during the COVID-19 pandemic. The following response options were available on a four-point Likert scale: ‘No, not uncertain at all’, ‘No, hardly uncertain’, ‘Yes, somewhat uncertain’, and ‘Yes, very uncertain’. For the statistical analyses, they were combined in the following two categories ‘not at all/hardly uncertain’ and ‘somewhat/very uncertain’.

The perceived comprehensibility of the rules against the spread of SARS-CoV-2 was captured by means of a question from the COVID-19 snapshot monitoring (COSMO) [22], a periodically online study relating to the risk perception and communication in Germany relating to the COVID-19 pandemic. The question, which was adapted for the telephone interview, was: ‘On a scale of 1 to 7, 1 means contradictory, and 7 means clear. With the values in-between, you can grade your response. For me, the current rules for the containment of the coronavirus are …’ [23]. For the calculation, three categories were created from the information on the seven-point Likert scale: Data with the values 1 and 2 formed the category ‘contradictory’, the values 3 to 5 formed the category ‘less clear’, and the values 6 and 7 form the category ‘clear’.

Determining whether the participants belonged to a risk group for a SARS-CoV-2 infection or for a severe course of the disease, respectively, was accomplished by querying disease-related risk factors known at the time of the survey in the following manner: ‘Next, we would like to know, to what extent you are part of a risk group for some infectious diseases. For this, I will read out several underlying diseases to you and when I am done, please tell me if you have one or several of the underlying diseases I mentioned. If you have none of the mentioned underlying diseases, please respond with ‘no’: Cardiovascular diseases, for example heart disease and high blood pressure; Chronic lung diseases, for example COPD; Chronic kidney and liver diseases; Diabetes mellitus, diabetes; Cancer; Severe mental disease, for example schizophrenia or severe depression; Weakened immune system, congenital or acquired; Obesity, severe overweight’. There were two response options: ‘Yes, I have one or several of the mentioned diseases.’ Or ‘No, I do not have any of the mentioned diseases.’.

The vaccination status was collected with the question ‘Did you get vaccinated against the coronavirus, also referred to as COVID-19?’. Those who specified either ‘yes, once’ or ‘yes, twice’, were considered to be vaccinated; The classification as ‘unvaccinated’ was made accordingly with the answer ‘no’.

### 2.3 Study population

The survey data originated from four waves (wave 3, 4, 6, and 7) of the COVIMO study, and was pooled for the analyses. The analyses were based on data of N=3,998 participants with valid information relating to the participation in preventive measures during the pandemic (women: n=2,149, men: n=1,828). 21 participants provided no information about the gender identity and were disregarded in the gender-based evaluations. The eight individuals who assigned themselves to the category ‘diverse’, could also not be included in gender-based evaluations due to the small number of cases. The analyses relating to the decreased participation in preventive measures for non-communicable diseases during the COVID-19 pandemic were based on data from a total of 1,632 participating individuals (women: n=1,038, men: n=586).

The calculations were made using a weighting factor, which was calculated for the analyses and which corrects deviations of the sample from the population structure (as of: 31.12.2020) with regard to sex, age, and education. The COVIMO sample was thereby divided into partial populations (strata), which do not overlap and for which the population figures were known. In the sample, the weights were changed in each stratum in such a way that the estimated number corresponds to the external information. The weighting was made iteratively according to the so-called ‘raking’ method [24]. To make the information from the participants relating to education comparable, the International Standard Classification of Education (ISCED) was used for the weighting, which is based on information about school-leaving and vocational qualifications [25]. A detailed description of the methodology of COVIMO can be found in the methodology report relating to the COVIMO study [21].

### 2.4 Statistical methods

To answer the questions about the participation or the decreased participation, respectively, in prevention and health promotion measures, the information from participants of the COVIMO study was considered descriptively and was examined for group differences using the Chi-square test. Relative frequencies were reported with 95% confidence intervals (95% CI). These are estimated values, the accuracy of which can be rated with the help of confidence intervals – broad confidence intervals suggest a larger statistical uncertainty of the results. The confidence intervals (CI) were determined on the Logit scale. A significant difference was assumed when the p-value calculated considering the weighting and the survey design was less than 0.05.

The connection between pandemic-related factors and a lower participation in prevention and health promotion measures was also estimated by means of logistic regression using odds ratios (OR). The odds ratio indicates the factor by which the statistical odds of a lower participation in one group is increased compared to a reference group. Pandemic-related variables were included in Model 1, and an adjustment by sociodemographic variables was additionally made in Model 2. The following categories were in each case used as reference group (Ref.) in the regression models: Uncertainty because of a large amount of information: Ref.: Not at all/hardly; Comprehensibility of rules: Ref.: Clear; Risk group for SARS-CoV-2 infection: Ref.: No; Vaccination status: Ref.: Vaccinated; Age group: Ref.: Aged 18–29, and education group: Ref.: High education group.

The analyses were made using SAS 9.4. To appropriately consider the weighting in the calculation of confidence intervals and p-values, all analyses were calculated using the survey procedures of SAS.

## 3. Results

### 3.1 Participation in prevention and health promotion measures

Almost two thirds of participants indicated that they generally do not use prevention and health promotion programmes in the form of, for example, courses, exercises, or counselling (63.1%). Just over one quarter (28.3%) reported a lower participation in the last 12 months. 6.5% of participants indicated an unchanged participation, and 2.1% utilised more programmes (Table 1). Therefore, a total of 36.9% used the measures in general.

There was a significant difference between the genders (p<0.001). The proportion of those who generally did not use these measures was significantly higher in men with 70.0% than in women with 56.6%, while 33.6% of women indicated a lower participation, but only 22.7% of men.

With regard to the sociodemographic characteristics age (p=0.005) and education (p=0.007), there were significant differences only within the group of women (Table 1): It was noticeable that only approximately half of the women aged between 45 and 64 generally did not participate in the programmes, while in the other age groups this was partially almost two thirds. At the same time, the 45- to 64-year-olds were the age group with the highest proportion for a lower participation in the last 12 months (41.5%). The low education group had the highest proportion of women who generally did not participate: 66.0% versus 53.8% (medium education group) and 52.8% (high education group), respectively. It was also noticeable that in the group with a higher participation, the proportion of young women aged between 18 and 29 and of women with a high education was above average.

### 3.2 Factors for a lower participation in prevention and health promotion measures

**Figure 1:**
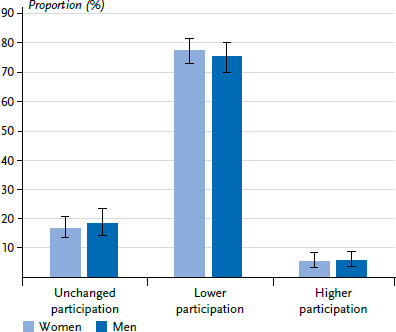
Participation in preventive measures during the COVID-19 pandemic 2020/2021 by women and men in the three population groups, which generally participate in such measures, relative frequency in percent (n=1,632, n=1,038 women, n=586 men) Source: COVIMO 2021 (pooled data of waves 3, 4, 6, 7)

Those participants who indicated that they generally do not use such programmes at all were initially excluded for the analysis of the factors that might impact a lower participation in the preventive measures. Of those who indicated that they used such programmes, the group with a lower participation in the last 12 months was by far the largest. In the pandemic years 2020 and 2021, more than three quarters of women and men reported a lower utilisation compared to approximately one sixth with an unchanged participation, and approximately 6% with a higher participation (Figure 1).

Different, pandemic-related factors were used to examine, which factors could be related with a lower participation in prevention and health promotion measures in the last 12 months (Table 2). Within the group of participants who used fewer programmes, the number of people who felt ‘not at all/hardly uncertain’ and the ones who felt ‘somewhat/very uncertain’ was approximately the same, that is, there were no significant differences with regard to the level of uncertainty on the basis of a large amount of information about the pandemic. The situation is different with the perceived comprehensibility of the rules relating to the pandemic. Men who perceived the existing rules for the containment of SARS-CoV-2 to be contradictory (74.1%) or to be less clear (82.8%) at the time of the survey, are more frequent in the group with a lower participation than those who classified the rules to rather be clear (62.1%). This significant difference could not be observed in women. Even though individuals who were, based on their information about various diseases, assigned to a risk group for an infection with SARS-CoV-2 or a severe course of the disease in the event of contracting COVID-19, more likely to report a lower participation than those without risk, but the difference was not significant. The same was the case with the vaccination status. Even though in the group with a lower participation, the group of unvaccinated individuals was larger than the group of vaccinated individuals, the difference was not significant, neither in women nor in men.

When examining the association between pandemic-related factors and a lower participation in prevention and health promotion measures in the last 12 months, sociodemographic factors were also considered. It was noticeable thereby that proportionately more women of the two higher age groups (aged 45–64 and aged 65 and over) indicated that they use the measures less than the younger group. The same could be observed for the low and medium education group, compared to the high education group. However, the differences are not significant. The distribution of the frequencies of the sociodemographic factors was exactly the opposite in men. The proportions of the two younger age groups (aged 18–29 and 30–44) among those who participate less were higher than the proportions of the older age group, and the high education group had the highest proportion compared to the two other education groups. These differences are also not significant (Table 2).

The regression analyses largely confirmed the bivariant results for a lower participation in prevention and health promotion measures in the last 12 months during the pandemic years 2020 and 2021. As can be seen in Figure 2a, the results for women suggest an impact of the four examined pandemic-related factors. In the regression model, which is adjusted by sociodemographic factors (Figure 2b), the calculated OR for the pandemic-related factors also consistently reach positive values (OR from 1.1 to 1.8), but the OR are not significant compared to the respective reference group.

For men, a similar picture emerges with regard to the connection between pandemic-related factors and a lower participation in the prevention and health promotion measures. As can be seen in Figure 3a and 3b, the results for men also point towards a possible association in three of the examined pandemic-related factors (uncertainty due to a large amount of information, risk group for SARS-CoV-2 infection, vaccination status). In the regression model, which is adjusted by sociodemographic factors (Figure 3b), almost all of the respective OR reached positive values between 1.5 and 2.0 (with the exception of the OR of 0.7 of the value of ‘uncertainty due to large amount of information about the pandemic’), but the OR are not significant compared to the respective reference group. In men, however, the regression calculations confirmed a pandemic-related factor, which is already significant in the bivariate analyses (Figure 3b): Men who perceived the rules relating to the pandemic to be less clear had ‘odds’ that were increased 3.3-times of participating less in the measures (compared to the group that rated the rules to be clear).

## 4. Discussion

In 2020 and 2021, the COVID-19 pandemic resulted in wide-scale restrictions in everyday life in Germany, which also hampered and temporarily hindered the implementation and utilisation of prevention and health promotion measures. Our analyses show, how this impacted the utilisation of prevention and health promotion for the prevention of non-communicable diseases.

Almost two thirds of the participants generally did not use the measures in the form of, for example, courses, exercises, or counselling in the last 12 months, 7% used the programmes to the same extent, and 2% even to an increased extent. Just over one quarter decreased the participation during that time. Gender, age, and education were associated with differences in participation. When only looking at the group, which generally uses these measures in the form of, for example, courses, exercises, or counselling, then it is even three quarters that participated more rarely in measures during the pandemic years 2020/2021.

In the population group with a decreased participation, various pandemic-related factors were examined with regard to an association. Even though differences could be observed, significant differences could be found within the group of men only for the perceived comprehensibility of the rules against the spread of SARS-CoV-2.

### 4.1 Classification of the results

The relatively high proportion of 36.9% that generally used the programmes is noticeable in the analyses in 2020/2021. This is more than double compared to the determined frequency for the period between 2008 and 2011 (16.6% in the German Health Interview and Examination Survey for Adults (DEGS, [17]) or 16.0% in the study German Health Update (GEDA) 2009 [26]), respectively, and approximately four times compared to 1997 to 1999, in which this question was raised with the German Health Interview and Examination Survey (BGS98) (9.1%, [17]). The results from these studies, however, only allow an approximate comparison because even though the population itself was surveyed there as well, slightly different formulations of questions and partly different survey modes were used, and the data analyses in these studies partly focused on individuals insured by statutory health insurance. However, current data from the prevention report of the statutory health insurance also point towards an increased utilisation until the start of the pandemic. The services of the statutory health insurance and the groups of people that are reached by primary prevention and health promotion in different settings and at the workplace are reported. Until the pre-pandemic year 2019, the proportion of companies/sites that have been reached by workplace health promotion (BGF) increased approximately 3.5-times since 2010 and the number of the other settings that were reached with health promotion measures increased about 1.5-times [6, p. 51, 71]. A significant proportion of behaviour-related measures especially on the topics of physical activity and diet were implemented thereby [6]. The expansion of the mere individual behaviour-based prevention remained approximately at a similarly high level [6, p. 98]. Overall, the conclusion based on the presented results that there was a further increase in the utilisation of prevention and health promotion measures in the form of courses, exercises, or counselling in the last ten years, seems plausible.

The differences relating to the participation frequency in 2020/2021, which were observed with the data from the COVIMO study, with regard to different sociodemographic groups, large coincide with the current state of research relating to the utilisation before the pandemic. The differences between women and men correspond to the insights from earlier research [6, 27–30], for example analyses with data from the studies DEGS [17] and GEDA [26], which found a participation in the prevention and health promotion measures significantly more frequently for women than for men [17, 26]. The generally observable higher health consciousness of women, and the measures that are generally not designed to be gender-specific, are considered to be cause and explanation for this difference [31]. The higher participation in the middle and higher age groups that can be observed within the group of women, also became apparent in studies, which examined the utilisation of prevention and health promotion at earlier points in time [17, 26]. Earlier studies for Germany likewise found these age differences in the group of men [26, 29, 31]. The health consciousness, which increases with increasing age, is also considered to be an explanation here for the utilisation, which rises with age [32]. The education differences, which were only observed for women, also became apparent in other studies, which found no significant or only slight differences between the education groups or social status groups, respectively, for men [17, 31]. These are indications of an interaction between the factors of gender and social status with regard to preventive or health-promoting behaviour, respectively [31, 33].

Three quarters of those who generally used these prevention and health promotion measures, decreased their participation during the pandemic in 2020/2021. The assumption that the pandemic-related factors examined here – uncertainty due to a large amount of corona-related information, comprehensibility of the rules for the containment of the pandemic, belonging to a risk group for a SARS-CoV-2 infection, vaccination status – are associated with a lower participation in the measures, was largely not confirmed in our data analysis. This allows drawing the conclusion that there are other factors, such as, for example, the containment measures, in particular the lockdown, but also closure of enclosed spaces that were in place in phases, and contact limitations, which led to a smaller offered range of such measures. This hindered women and men who generally participate in the measures, from actually using them during the pandemic. Thus, it failed to reach at least vulnerable groups, such as individuals with a social disadvantage who have a higher risk for non-communicable diseases, but also for an infection with SARS-CoV-2 or a severe course of COVID-19, with these health promotion measures.

In our reported results about possible factors, which could be associated with a lower participation in health promotion measures in the pandemic years 2020/2021, a significant association became apparent for the group of the men only for the perceived comprehensibility of the rules against the spread of SARS-CoV-2. If men perceived the rules to be less clear, they used fewer measures. The COSMO study was able to show that individuals who are more familiar with the current rules, perceive them to be less contradictory than individuals who are less familiar with them [20]. This could mean that men who were less familiar with the rules were more uncertain or had less information about how they could have participated in the programmes that still existed or in alternatives, for example digital programmes, or programmes outdoors. Gender-specific differences in the search for health information were already known before the pandemic. Men look for health information less frequently than women [34, 35]. It was shown during the COVID-19 pandemic, for example, that men used online media less frequently to look for information than women during the lockdown [36]. Gender-specific differences should be taken into consideration for the communication in crisis situations, e.g. for the communication of pandemic-related information, such as the currently applicable rules.

The gender-related and social differences confirmed with the COVIMO data for the general participation in prevention and health promotion measures have already been known for approximately two decades. In spite of the utilisation, which increased overall during this period, it is still more difficult to reach men and people from the low education group with prevention and health promotion measures for the prevention of non-communicable diseases. This problem, which is referred to as prevention dilemma, represents one of the biggest challenges for public health in Germany and became even more relevant during the COVID-19 pandemic. Socially disadvantaged population groups generally have a higher health burden caused by non-communicable diseases [37] and were more severely affected by SARS-CoV-2 infections [38] and psychosocial effects [14] over the course of the pandemic. Accordingly, existing socially-induced health inequalities increased during the pandemic, which was not only observed in Germany, but also in other countries [39]. For prevention and health promotion in Germany, this means offering pandemic-specific support on the one hand [15, 16]. The switch to or enhancement by digital programmes, respectively, can only be one measure thereby [6] because even though socially disadvantaged population groups use digital media just as frequently as other groups, they benefit less therefrom (third-level digital divide) [40]. In a survey of 98 health insurance funds and associations of health insurance funds conducted in 2021, they indicated that vulnerable groups are difficult to reach and decreasing equal health opportunities due to the pandemic [41]. On the other hand, structures and conditions should be created, which make it possible even in times of crisis, such as the pandemic, to maintain prevention and health promotion measures. These necessary ‘resilient structures for health promotion’ need to be organised and equipped so that they provide for creativity and flexibility in order to cope with unforeseeable conditions [6, p. 14, 41] and simultaneously promote the equal health opportunities [42]. For future protective measures in the COVID-19 pandemic, other epidemics, or social crises, this means planning health promotion and prevention for non-communicable diseases alongside the development of the containment measures, and taking social determinants in terms of the health-in-all-policies approach into consideration [43].

### 4.2 Strengths and weaknesses

The presented results are not only the first set of data concerning the utilisation of prevention and health promotion measures during the pandemic in 2020/2021, but generally the first set of data in a long time concerning the participation of adults in these programmes from the population’s perspective. The analyses provide important information about the extent of the measures in the pandemic years 2020/2021 and take the significance of sociodemographic, but also of pandemic-related factors, into consideration.

When interpreting these results, it is important to take into consideration that the survey period was from 17.3.2021 until 18.8.2021 and thus covered a relatively large time period. Due to the fact that the participants were to base their response relating to the participation in prevention and health promotion measures on the last 12 months, it becomes clear that the participants based their responses on different periods. With regard to the course of the pandemic, these were periods with varying degrees of restrictions. It is also important to keep in mind that the vaccination status used in the analyses does take into consideration, how long ago the interviewed individual got vaccinated. This limits the interpretation of the factor of vaccination status to a lower participation in the measures because the participation was based on the last 12 months.

Last but not least, it is important to point out that, when assessing the results, this is a cross-sectional study, and that the results represent associations, but cannot reveal any causations. In addition, it is important to consider the structure of the sample. Only German-speaking individuals who could be reached by telephone either on their mobile phone or via a landline, were interviewed for the COVIMO waves used here. It is thus possible that small subgroups, which may be particularly vulnerable, were not reached.

The results of the study suggest additional need for research. With regard to a lower participation in the measures, the pandemic-related factors examined in this study, which did not reveal any significant differences with very large confidence intervals, should be examined once again in larger samples. Additional pandemic-related factors could be used thereby. These include structural determinants, for example the availability of programmes, but also individual factors, such as the risk perception and the attitude towards and the handling of SARS-CoV-2 protective measures. They were used, for example, in the COSMO study [23], but could not be examined here. For the future communication under pandemic conditions, it would also be important to know, how these determinants need to be worded in a target group-specific manner, in order to motivate especially population groups with a higher risk for non-communicable diseases to utilise prevention and health promotion measures even during a crisis situation.

### 4.3 Conclusion

Fortunately, the use of prevention and health promotion measures to prevent non-communicable diseases seems to have increased over the last ten years. The pandemic stopped this development. Hopefully, this development was only interrupted and will reach a pre-pandemic level in the next few years again. It was also more difficult to reach socially disadvantaged individuals during the pandemic years 2020/2021. Resilient structures, which provide measures for reaching disadvantaged groups and which counteract the social health inequalities, even in times of crisis, are thus required. Due to the fact that prevention and health promotion measures for non-communicable diseases have the potential to at least partly counteract the psychosocial and health consequences of the crisis during a pandemic situation, they should be part of the crisis planning in epidemically significant situations in the future.

## Key statements

- 28% of participants used fewer prevention and health promotion measures in 2020/2021, 7% used them just as often, and 2% used more. Almost two thirds generally did not use these measures in general.
- Men reported significantly more frequently than women that they generally do not participate in prevention and health promotion measures.
- Women in the middle age group or with medium or high education used prevention and health promotion measures more frequently than the respective comparison groups.
- A lower participation was only associated with the pandemic-related factor ‘perceived comprehensibility of the rules against the spread of SARS-CoV-2’ among men.
- Health promotion and prevention of non-communicable diseases should be part of contingency planning in epidemically significant situations.

## Figures and Tables

**Figure 2a:**
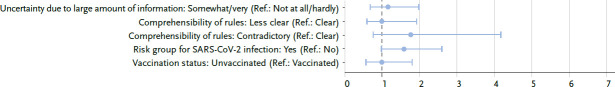
Associations between a lower participation in preventive measures during the Corona pandemic 2020/2021 and pandemic-related factors, women, odds ratios (n=1,038) Source: COVIMO 2021 (waves 3, 4, 6, 7)

**Figure 2b:**
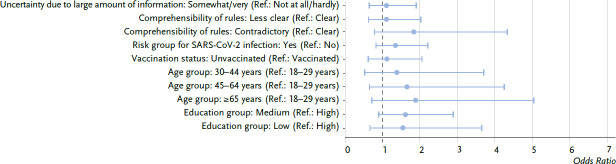
Associations between a lower participation in preventive measures during the Corona pandemic 2020/2021 and pandemic-related factors, women, odds ratios, adjusted by sociodemographic factors (n=1,038) Source: COVIMO 2021 (waves 3, 4, 6, 7)

**Figure 3a:**
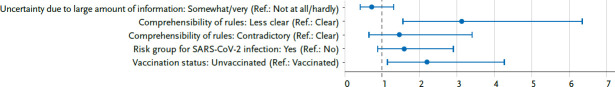
Associations between a lower participation in preventive measures during the Corona pandemic 2020/2021 and pandemic-related factors, men, odds ratios (n=586) Source: COVIMO 2021 (waves 3, 4, 6, 7)

**Figure 3b:**
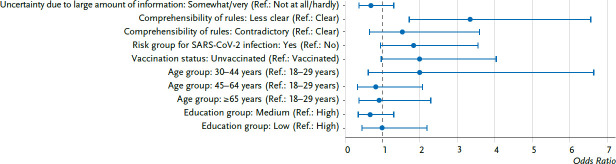
Associations between a lower participation in preventive measures during the Corona pandemic 2020/2021 and pandemic-related factors, men, odds ratios, adjusted by sociodemographic factors (n=586) Source: COVIMO 2021 (waves 3, 4, 6, 7)

**Table 1:** Participation in preventive measures during the COVID-19 pandemic 2020/2021 by women and men by age and education, relative frequency in percent (total N=3,998, women n=2,149, men n=1,828) Source: COVIMO 2021 (pooled data of waves 3, 4, 6, 7)

	Generally no participation (n=2,366)		Unchanged participation (n=313)		Lower participation (n=1,234)		Higher participation (n=85)	
	%	(95% CI)	%	(95% CI)	%	(95% CI)	%	(95% CI)
Total (women and men)^*^	63.1	(60.7-65.5)	6.5	(5.4-7.8)	28.3	(26.1-30.6)	2.1	(1.5-2.9)
**Women (total)**	**56.6**	**(53.2–60.0)**	**7.3**	**(5.7–9.3)**	**33.6**	**(30.6–36.8)**	**2.4**	**(1.5–3.8)**
Age group^*^								
18–29 years	60.5	(49.4–70.6)	7.1	(3.4–14.3)	27.0	(18.2–38.1)	5.3	(1.9–14.3)
30–44 years	62.6	(54.4–70.2)	9.3	(5.5–15.3)	27.1	(20.7–34.7)	1.0	(0.2–3.7)
45–64 years	47.8	(42.7–53.0)	7.4	(4.9–11.1)	41.5	(36.4–46.7)	3.3	(1.8–6.0)
≥65 years	60.0	(55.0–64.8)	5.9	(4.1–8.3)	33.1	(28.6–37.9)	1.0	(0.5–2.0)
Education status (schooling)^*^								
Low education group	66.0	(58.9–72.4)	5.8	(2.9–11.2)	27.8	(22.0–34.4)	0.5	(0.1–1.9)
Medium education group	53.8	(48.2–59.3)	6.3	(4.2–9.4)	37.3	(32.1–42.8)	2.5	(1.2–5.1)
High education group	52.8	(48.0–57.5)	10.2	(7.4–13.8)	33.1	(28.9–37.6)	3.9	(2.0–7.5)
**Men (total)**	**70.0**	**(66.5–73.2)**	**5.6**	**(4.2–7.3)**	**22.7**	**(19.7–26.0)**	**1.8**	**(1.1–2.7)**
**Age group**								
18–29 years	67.8	(56.9–77.0)	5.4	(2.4–11.6)	24.5	(15.9–35.8)	2.4	(0.9–6.1)
30–44 years	73.2	(65.2–79.9)	3.1	(1.4–6.8)	22.7	(16.4–30.6)	1.1	(0.3–3.6)
45–64 years	68.5	(62.9–73.6)	7.2	(4.7–11.1)	22.4	(18.1–27.5)	1.9	(0.9–3.7)
≥65 years	70.7	(64.9–75.8)	5.6	(3.8–8.1)	21.9	(17.2–27.4)	1.9	(0.8–4.4)
**Education status (schooling)**								
Low education group	70.4	(62.4–77.3)	5.8	(3.0–11.2)	21.7	(15.5–29.4)	2.1	(0.8–5.1)
Medium education group	70.0	(63.7–75.6)	5.5	(3.6–8.4)	22.3	(17.1–28.5)	2.2	(1.2–4.2)
High education group	69.1	(64.7–73.2)	5.4	(3.7–7.9)	24.3	(20.6–28.5)	1.1	(0.6–2.1)

CI = confidence interval

^*^ = significant with p<0.05

**Table 2:** Pandemic-related and sociodemographic factors in individuals with lower participation in preventive measures during the COVID-19 pandemic 2020/2021 by gender, relative frequency in percent* (n=1,632) Source: COVIMO 2021 (pooled data of waves 3, 4, 6, 7)

	Women (n=1,038)			Men (n=586)		
	%	(95% CI)	p-value	%	(95% CI)	p-value
Total	77.6	(72.9–81.6)		75.5	(69.8–80.5)	
**Pandemic-related factors**						
**Uncertainty due to large amount of information**			0.373			0.962
Not at all/hardly uncertain	75.6	(69.6–80.7)		75.6	(68.1–81.7)	
Somewhat/very uncertain	79.6	(72.0–85.6)		75.3	(65.6–83.0)	
**Comprehensibility of the rules**			0.313			**0.009**
Contradictory	83.8	(74.2–90.3)		74.1	(61.8–83.5)	
Less clear	75.8	(69.1–81.5)		82.8	(76.1–87.8)	
Clear	76.3	(66.8–83.7)		62.1	(48.1–74.4)	
**Risk group for SARS-CoV-2 infection**			0.102			0.405
Yes	81.8	(75.1–87.0)		78.0	(71.0–83.7)	
No	74.6	(68.0–80.1)		73.6	(64.6–81.0)	
**Vaccination status**			0.617			0.059
Vaccinated	76.7	(70.6–81.8)		70.0	(62.1–76.8)	
Unvaccinated	79.0	(71.4–85.0)		80.4	(71.9–86.8)	
**Sociodemographic factors**						
**Age group**			0.191			0.339
18–29 years	68.4	(50.2–82.3)		76.0	(58.6–87.6)	
30–44 years	72.6	(59.7–82.6)		84.7	(71.8–92.3)	
45–64 years	79.5	(72.3–85.1)		71.1	(61.0–79.5)	
≥65 years	82.8	(77.1–87.3)		74.6	(65.3–82.0)	
**Education status (schooling)**			0.086			0.665
Low education group	81.6	(68.7–89.9)		73.3	(58.3–84.3)	
Medium education group	80.9	(73.9–86.3)		74.1	(64.4–82.0)	
High education group	70.2	(62.5–76.8)		78.8	(71.6–84.5)	

^*^Based on the population groups that generally participate in such measures; Comparison group: combined proportions of unchanged and higher participation Bold: Significant with p<0,05, CI = confidence interval
